# Proximal Femoral Morphology and the Relevance to Design of Anatomically Precontoured Plates: A Study of the Chinese Population

**DOI:** 10.1155/2014/106941

**Published:** 2014-11-03

**Authors:** Kun-Jhih Lin, Hung-Wen Wei, Kang-Ping Lin, Cheng-Lun Tsai, Pei-Yuan Lee

**Affiliations:** ^1^Translation Technology Center for Medical Device, Chung Yuan Christian University, Zhongli 32023, Taiwan; ^2^Department of Physical Therapy and Assistive Technology, National Yang-Ming University, Taipei 11221, Taiwan; ^3^Department of Electrical Engineering, Chung Yuan Christian University, Zhongli 32023, Taiwan; ^4^Department of Biomedical Engineering, Chung Yuan Christian University, Zhongli 32023, Taiwan; ^5^Department of Orthopedics Surgery, Show Chwan Memorial Hospital, No. 542, Chung-Shang Road, Zhongli 500, Taiwan

## Abstract

Adequately shaped femoral plate is critical for the fixation of fracture in the pertrochanteric regions. Lateral aspect of greater trochanter is an important region where the proximal femoral plate anchored. However, little is known regarding the morphology of greater trochanter. The objective of this study was to measure main dimensions of greater trochanter and other regions in the proximal end of the femur to provide an anatomical basis for the design of the proximal femoral plate. Anthropometric data on the proximal femur were performed utilizing three-dimensional computational modeling. Computed tomography images of healthy femurs in 53 women and 47 men were contributed to three-dimensional femur modeling. All data were compared between male and female femora. The results showed that mean values for male group were found to be greater in most of measured femoral dimensions. Oppositely, females demonstrated higher neck-shaft angle on anteroposterior view and femoral anteversion angle. The anthropometric data can be used for the anatomical shape design of femoral plates for osteosynthesis of fractures in the trochanteric regions. A distinct plate design may be necessary to accommodate differences between the genders.

## 1. Introduction

Intertrochanteric and subtrochanteric fractures are major pathology in all fractures of the proximal femur [1]. Several devices were developed to treat these types of fractures including the dynamic hip screw, 95° angled blade plates, and intramedullary nails, but fixation failures such as varus collapse of femoral head and cut-out of the proximal femoral screw were frequently reported [2–4]. In 2007, the proximal femoral-locking compression plate 4.5/5.0 (PF-LCP, Synthes, West Chester, PA, USA), a new precontoured and fixed-angle fixation device, was introduced to improve osteosynthesis of pertrochanteric fracture of the femur. However, recent clinical results had showed high failure rates of trochanteric fracture fixation with the proximal femoral-locking compression plate [5–8].

The goal of surgical intervention is to achieve anatomical reduction with a stable fracture fixation which helps bone union and allows early mobilization. From the biomechanical viewpoint, a better contour fit between bone and plate is crucial to establish a stronger bone-plate construction and to stabilize the fracture fragments even with a locking compression plate [9] and may facilitate an appropriate placement of the plate intraoperatively which saves time and prevents improper position of the proximal screw within femoral head because the screw is locked in the plate. Lateral roentgenogram of the proximal femur revealed a posterior bow of the metaphysis and an anterior bow of the diaphysis [10]. With a straight typed proximal femur plate, it may be difficult for the surgeons to determine a suitable plate placement, thus leading to incorrect proximal screw position and subsequent screw cut-out [5, 6]. Furthermore, lateral approach is a standard for the plate passed through the greater trochanter. Shape of the plate can be designed with respect to the three-dimensional morphology of the greater trochanter for an easy and adequate implant placement; otherwise malposition of the plate would alter its biomechanical behavior and weaken the capacity of mechanical stability for injured bone [11]. Therefore, understanding the geometry of the greater trochanter is very important for either surgical operation or implant design.

To our knowledge, no anthropometric research exists evaluating the morphology of the proximal end of the femur, especially the greater trochanter. The objective of this study was to measure the geometric parameters of the CT-based proximal femur models to provide a basis for an anatomical design of the extramedullary proximal femoral plate. Additionally, comparison of anatomical parameters between the genders was performed also. We used three-dimensional (3D) computational modeling to pursue quantitative understanding of the proximal end of the femur.

## 2. Materials and Methods

Totally, 100 femur computed tomography (CT) scans done in 100 selected participants at Show Chwan Memorial Hospital were evaluated and the study was approved by the Institutional Review Board, Show Chwan Memorial Hospital (SCMH_IRB number 1010705). We included subjects of more than 20 years of age with nonpathologic and normal low limb alignment bones and specifically excluded those with hip joint arthritis or historical injury and bony deformity. A prospective study of 100 normal subjects was conducted under computer tomography scanning. Fifty-three women and 47 men were involved with right femur analyzed. The CT scans (Light Speed VCT, GE Medical System, General Electric Company, USA) were collected with slice thickness of 1.25 mm and 512 × 512 pixels per image. The subjects were scanned for CT in a supine position with neutral rotation of the low limb. A complete femur segmentation from proximal to distal ends was acquired and each image was obtained in Digital Imaging and Communications in Medicine (DICOM) format. The CT scan data were then imported into self-development imaging software to outline cortical contour by using thresholding technique. PTC Creo 2.0 (Parametric Technologies Corp., Needham, MA, USA), a CAD software, was utilized to reconstruct the three-dimensional bony model via the cortical contours and 3D volume rendering and subsequently geometric measurements. Each 3D femoral model was rotated to the true anteroposterior (AP) and lateral viewings, confirmed by a senior orthopaedic surgeon (P.-Y. Lee). All measurements were carried out by the built-in commands of PTC Creo.

### 2.1. Measurement of the Anatomical Parameters from AP View

**Figure 1:**
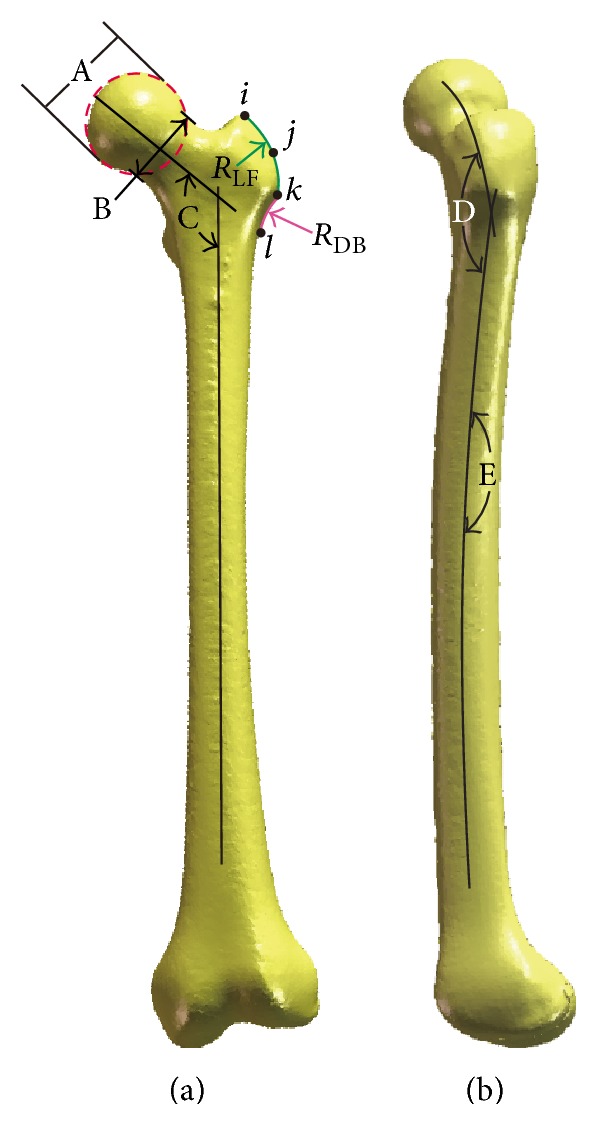
Measurements of femoral dimensions taken from (a) AP view and (b) lateral view. A: femoral head diameter; B: narrowest neck width; C: neck-shaft angle (NSA); D: sagittal NSA; E: medullary radius of curvature.

In order to evaluate the shape of the greater trochanter, four points on the lateral aspect of the greater trochanter were established. These four points were proximal apex (*i*), lateral apex (*k*), the middle (*j*) between the proximal and lateral apexes, and the base of the greater trochanter (*l*). The shape of the greater trochanter was then evaluated across these four points. Following morphometric parameters were obtained (Figure 1(a)): (1) neck-shaft angle (NSA), angle between the axis of the femoral neck and the femoral shaft, (2) femoral head diameter, (3) narrowest neck width, (4) the superior radius, radius of a best-fit circle fitted to three points (*i*, *j*, and *k*) on the lateral facet of the greater trochanter (*R*_LF_), and (5) the inferior radius, radius of a best-fit circle fitted to two points (*k* and *l*) on the distal border of the greater trochanter (*R*_DB_).

### 2.2. Measurement of the Anatomical Parameters from Lateral View

**Figure 2:**
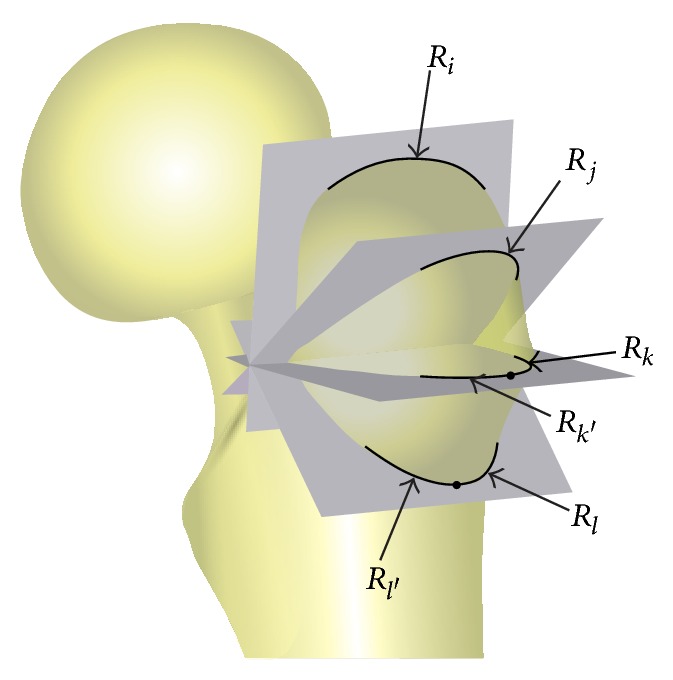
Diagram of four planes established, which were virtual cross sections and included the points *i*, *j*, *k*, and *l*, respectively. The radius of the intersected curve between each plane and the greater trochanter was then measured to evaluate the shape of the greater trochanter. Each radius measured is shown (*R*_*i*_, *R*_*j*_, *R*_*k*_, *R*_*k*′_, *R*_*l*_, and *R*_*l*′_).

Medullary radius of curvature of the femur (anterior bowing) and a specially defined neck and shaft angle (sagittal NSA) were measured. The medullary radius of curvature was calculated by three-point circle algorithm where the three points were identified as the middle points of 3 lines drawn across the medullary canal from proximal to distal. As for the sagittal NSA, the posterior curve and the anterior curve were defined, which describe the posterior bow of the metaphysis and the anterior bow of the diaphysis, respectively. The angle of intersection of these anterior and posterior axes is the sagittal NSA (Figure 1(b)). In addition, we established four planes through four points (*i*, *j*, *k*, and *l*) on the lateral aspect of greater trochanter as the virtual cross sections including the intersection of femoral neck axis and femoral shaft axis, respectively. Then, the radius of the curve of each intersected outline was extracted (*R*_*i*_, *R*_*j*_) (Figure 2). If single radius cannot represent the intersected outline properly, two radii would be drawn out (*R*_*k*_, *R*_*k*′_, *R*_*l*_, and *R*_*l*′_).

Also, the anteversion of femoral neck was derived from determining the angle between the transcondylar plane and the plane constructed by the intersection of the axis of the neck and the axis of the femoral shaft [12].

### 2.3. Statistical Analysis

The data were summarized as the mean and standard deviation. Statistical analyses for comparison of morphologic features between males and females were done using unpaired *t*-tests or chi-square analysis. A *P* value of < 0.05 was indicated as statistically significant. The statistical package used was Microsoft Excel and its statistical software (Microsoft Corporation, Redmond, WA).

## 3. Results

The subjects' demographic data are shown in Table 1. There were no statistical differences between the genders in terms of age and side of femur analyzed in this study. Each of the femoral dimensions and angles measured is summarized in Tables 2 and 3 for the total group and for comparison between males and females.

### 3.1. Parameters from AP View

The mean neck-shaft angle, the mean femoral head diameter, the mean narrowest neck width, the mean superior radius of greater trochanter, and the mean inferior radius of greater trochanter of all subjects were 129.88°, 45.4 mm, 31.91 mm, 28.77 mm, and 64.90 mm, respectively (Table 2). For gender comparison, the male group had significantly greater values in all measured dimensions (*P* < 0.01) except the neck-shaft angle (*P* = 0.646).

### 3.2. Parameters from Lateral View

The mean section radii of *R*_*i*_, *R*_*j*_, *R*_*k*_, *R*_*k*′_, *R*_*l*_, and *R*_*l*′_, the mean medullar radius of curvature of the femur, the mean sagittal NSA, and the mean anteversion of all subjects were 24.02 mm, 18.44 mm, 33.91 mm, 19.89 mm, 49.73 mm, 18.99 mm, 930.13 mm, 170.66°, and 21.58°, respectively (Table 3). For all average section radii, the male group had significantly greater values (*P* < 0.05). Males in our sample also had on average greater medullar radius of curvature and the sagittal NSA (*P* < 0.01). For the femoral anteversion, the males had smaller angle of 11.66° compared with the females with a mean angle of 29.03° (*P* < 0.0001).

## 4. Discussion

Anatomical shape design of the proximal femoral plate is crucial to insert the plate readily, to conform to the metadiaphyseal bridging areas of the femur, and to avoid damaging the periosteum or the soft tissue [13]. To define the anatomical shape of the extramedullary fixator for the proximal femur, a comprehensive understanding of the bony morphology is crucial. However, previous morphologic studies focused on the femoral neck geometry or the medullary shape [14–17]. There are no medical literatures regarding the anthropometric data of the region around greater trochanter. The PF-LCP, a new device for treating fractures of the trochanteric region, claimed that the proximal portion of the plate is anatomically precontoured for the lateral aspect of the proximal femur [18], but it seems to lack the evident data of how the anatomical contour of the plate was developed. In the current study a geometric measurement of the proximal femur was carried out, specifically the greater trochanteric bald spot and adjacent region where the proximal portion of the plate anchored.

Among parameters measured in our study, the measured radii on the greater trochanter can be important reference for designing the proximal portion of the femoral plate. An anatomical proximal femoral plate design should not only have an anatomical contour but the ventral surface of the plate probably has to approximate the outer surface of the cortex to prevent a ventral gap from compromising the mechanical stability of the locking plate. Once the plate is fixed at an increased distance from the bone, the unsupported free part of the screw between the plate and the bone increases, leading to a greater lever arm effect. The resulting augmented bending moment weakens the bone-plate construct and results in screw loosening, backing-out, or breakage [5–7]. Therefore, understanding the curvature of the greater trochanter may be beneficial for better bone-plate fitness when designing the implant.

Femoral anteversion and the specially defined sagittal NSA could also be key parameters for the design of the proximal portion of the femoral plate and for the angulation or placement of the proximal screws during surgery. Femoral anteversion is a normal torsion or twist presented in the femoral neck. Accordingly, we thought that a twisted profile in the proximal portion of the femoral plate may be needed to correspond with the natural anteversion of the neck and an appropriate placement of proximal screws in the femoral head. Generally, there is an agreement concerning the fact that an exact central placement of the proximal screw on the lateral view is a recommended position [19] to prevent cut-out of the proximal screw [7, 20, 21]. Consequently, the twisted profile of the proximal portion of the femoral plate may accommodate the inherent torsion of the femoral neck and ensure a satisfactory proximal screw position without large margin of error. Specifically, our results showed that the mean angle of femoral anteversion for the female subjects (29.03°) was significantly higher than the male subjects (11.66°). Anthropometric studies also demonstrated that females were significantly more anteverted, irrespective of the differences among various ethnic groups [12, 22, 23]. These findings strengthen the necessity of a twisted design in the proximal portion of the femoral plate or different proximal screw angulation.

As for the medullary radius of curvature of the femur (anterior bowing), it may be a design consideration for the plate shaft but may not be involved in contemporary proximal femoral plates yet. At present, most long bone plates on market are straight type. However, there is a physiological anterior bowing of the femoral diaphysis. If the proximal femoral plate can be developed to most closely replicate the anatomical shape of the femur, like the commercialized clavicle plates which are anatomical clavicular plating system to match the S-shaped curvature of the clavicle [24], it would be helpful to maximize mechanical support, to accurately reduce the fracture, and to restore the patient's original anatomy.

The mean medullary radius of curvature of the femur was 930.13 mm in our 100 Chinese femurs. This value is similar to that previously reported by Tang et al. [25] (926.2 mm) whose samples were Chinese as well. However, Harper and Carson [26] (1140 mm) and Egol et al. [27] (1200 mm) all reported greater values of Caucasian than this study and Tang et al's measurement. This ethnic difference had been discussed and contributed to the development of the proximal femoral nail antirotation-Asian version (PFNA-II), a modification of the original PFNA [28]. Likewise, we thought that the precontoured shape in the shaft of the proximal femoral plate may have to be divided into different radii of curvature to adapt to the ethnic difference and to obtain best geometry fit.

Factors affecting the morphology of the proximal femur rely on genetic and environmental variations including age, race, sex, and lifestyle. Studies on the distal femur suggested significant differences in dimension and shape among the males and the females [29, 30]. Their findings implied that there may be a necessity of distinct design of implants to accommodate difference between the genders and other morphologic differences around the knee joint. Similarly, our results also found that there was significant difference between the genders with regard to the geometric parameters of the greater trochanter and other dimensions in proximal femur. This indicated that designing the proximal femoral plate may have to take the gender difference into consideration because the best solution is not shaping the implant to fit on the average anatomical contour of the population but is the one that provides best fit on the majority of the femora.

This study utilized 3D modeling method for femoral morphology measurement. 3D modeling of the proximal femur from CT has the advantage of obtaining the full shape of bones, as well as obtaining the anatomic landmarks. Relatively, images captured by two-dimensional methods, such as radiography, provide only a projection of bone shape and create a pattern of variability due to variations in the angle of the X-ray beam and magnification. However, because 3D rendering of the femur model is based on cortical contours outlined by image thresholding technique, this may generate certain femur shape variability due to the uncertainty of the optimized thresholding value.

Several limitations should be notified in the present study. One limitation is that only Chinese populations were included, but it does not invalidate the importance of our measurements and findings because of no greater trochanteric parameters available for the basis of the proximal femoral plate design. Another limitation is that it is not known if there is any difference in major dimensions of the greater trochanter in different racial groups. Further anthropometric study on the western population would be necessary to clarify any difference of greater trochanteric parameters between the Caucasian and the Chinese people. On the other hand, we did not take the third trochanter into consideration, which may be an influencing factor on femoral morphology. However, the incidence of the third trochanter varies as a result of the fact that incongruence of trochanter definition exists between studies [31]. Furthermore, Bolanowski et al. [31] concluded that the third trochanter was not correlated with any morphological feature of the femoral head, neck, and shaft although femora with the third trochanter showed a better developed greater trochanter than femora without the third trochanter. Consequently, this study evaluated only the effect of sexual difference on femoral anatomies. Finally, all the subjects involved were skeletal mature adults. The results should not be appropriate for the pediatrics [32, 33]. And, except sex, several other factors are quoted which affect femoral morphology: age, activities, genetic factors, body weight, muscle action, lifestyle, and disease [32, 34]. The correlation between these factors is still unknown.

In conclusion, the results of this study provided an anatomical data of the proximal end of the femur, especially in the region of greater trochanter. These femoral dimensions can be applied to the modifications of the contemporary femoral plate or a newly development for the Chinese population. The statistics analysis also revealed significant differences in dimensions between male and female femora, indicating that distinguished designs for the genders may be required to improve bone-implant fitness as discussed in the femoral fixation devices.

## Figures and Tables

**Table 1:** Subject demographics (cm; kg).

Parameters	Total	Men	Women
	(Mean ± SD)	(Mean ± SD)	(Mean ± SD)
Number	100	47	53
Age	36.47 ± 11.46	36.92 ± 11.90	36.14 ± 11.11
Height	164.08 ± 7.91	170.84 ± 5.91	158.98 ± 4.80
Weight	64.84 ± 12.97	73.82 ± 10.84	58.08 ± 10.01

**Table 2:** Femoral measurements from AP view (degree; mm).

Parameters	Total	Men	Women	*P* value
	(Mean ± SD)	(Mean ± SD)	(Mean ± SD)	
Neck-shaft angle	129.88 ± 5.76	129.59 ± 5.81	130.05 ± 5.64	0.646
Femoral head diameter	45.40 ± 3.21	48.04 ± 2.87	42.21 ± 2.49	<0.0001
Narrowest neck width	33.91 ± 4.38	35.71 ± 3.27	31.2 ± 2.80	<0.0001
Superior radius (*R*_LF_)	32.26 ± 3.09	33.32 ± 2.33	30.23 ± 3.67	<0.0001
Inferior radius (*R*_DB_)	58.90 ± 5.1	59.41 ± 5.74	57.81 ± 4.65	<0.01

**Table 3:** Femoral measurements from lateral view (mm; degree).

Parameters	Total	Men	Women	*P* value
	(Mean ± SD)	(Mean ± SD)	(Mean ± SD)	
Section radius on *i* (*R*_*i*_)	24.02 ± 2.31	28.01 ± 1.94	21.48 ± 2.06	<0.0001
Section radius on *j* (*R*_*j*_)	18.44 ± 1.23	19.22 ± 1.32	17.88 ± 1.02	<0.05
Section radius on *k* (*R*_*k*_)	33.91 ± 3.56	38.34 ± 2.36	31.06 ± 3.21	<0.0001
Section radius on *k* (*R*_*k'*_)	19.89 ± 1.41	22.50 ± 1.65	17.96 ± 1.07	<0.01
Section radius on *l* (*R*_*l*_)	49.73 ± 2.14	51.03 ± 3.25	46.84 ± 4.33	<0.01
Section radius on *l* (*R*_*l'*_)	18.99 ± 1.22	20.14 ± 1.89	18.84 ± 1.37	<0.05
Medullary radius of curvature	930.13 ± 117.34	974.47 ± 98.36	897.46 ± 229.87	<0.0001
Sagittal NSA	170.66 ± 10.31	172.86 ± 8.36	169.57 ± 9.89	<0.01
Anteversion	21.58 ± 3.32	11.66 ± 1.55	29.03 ± 2.73	<0.0001

NSA: neck-shaft angle.

## References

[1] Koval K. J., Zuckermann J. D. (2006). *Handbook of Fractures*.

[2] Suckel A. A., Dietz K., Wuelker N., Helwig P. (2007). Evaluation of complications of three different types of proximal extra-articular femur fractures: differences in complications, age, sex and surviving rates. *International Orthopaedics*.

[3] Sadowski C., Lübbeke A., Saudan M., Riand N., Stern R., Hoffmeyer P. (2002). Treatment of reverse oblique and transverse intertrochanteric fractures with use of an intramedullary nail or a 95° screw-plate: a prospective, randomized study. *Journal of Bone and Joint Surgery—Series A*.

[4] Madsen J. E., Næss L., Aune A. K., Alho A., Ekeland A., Strømsøe K. (1998). Dynamic hip screw with trochanteric stabilizing plate in the treatment of unstable proximal femoral fractures: a comparative study with the gamma nail and compression hip screw. *Journal of Orthopaedic Trauma*.

[5] Wirtz C., Abbassi F., Evangelopoulos D. S., Kohl S., Siebenrock K. A., Krüger A. (2013). High failure rate of trochanteric fracture osteosynthesis with proximal femoral locking compression plate. *Injury*.

[6] Streubel P. N., Moustoukas M. J., Obremskey W. T. (2013). Mechanical failure after locking plate fixation of unstable intertrochanteric femur fractures. *Journal of Orthopaedic Trauma*.

[7] Wieser K., Babst R. (2010). Fixation failure of the LCP proximal femoral plate 4.5/5.0 in patients with missing posteromedial support in unstable per-, inter-, and subtrochanteric fractures of the proximal femur. *Archives of Orthopaedic and Trauma Surgery*.

[8] Glassner P. J., Tejwani N. C. (2011). Failure of proximal femoral locking compression plate: a case series. *Journal of Orthopaedic Trauma*.

[9] Ahmad M., Nanda R., Bajwa A. S., Candal-Couto J., Green S., Hui A. C. (2007). Biomechanical testing of the locking compression plate: when does the distance between bone and implant significantly reduce construct stability?. *Injury*.

[10] Noble P. C., Alexander J. W., Lindahl L. J., Yew D. T., Granberry W. M., Tullos H. S. (1988). The anatomic basis of femoral component design. *Clinical Orthopaedics and Related Research*.

[11] Khalafi A., Curtiss S., Hazelwood R. A. S., Wolinsky P. (2006). The effect of plate rotation on the stiffness of femoral LISS: a mechanical study. *Journal of Orthopaedic Trauma*.

[12] Hoaglund F. T., Low W. D. (1980). Anatomy of the femoral neck and head, with comparative data from caucasians and Hong Kong Chinese. *Clinical Orthopaedics and Related Research*.

[13] Frigg R., Appenzeller A., Christensen R., Frenk A., Gilbert S., Schavan R. (2001). The development of the distal femur Less Invasive Stabilization System (LISS). *Injury*.

[14] Casper D. S., Kim G. K., Parvizi J., Freeman T. A. (2012). Morphology of the proximal femur differs widely with age and sex: relevance to design and selection of femoral prostheses. *Journal of Orthopaedic Research*.

[15] Karlsson K. M., Sernbo I., Obrant K. J., Redlund-Johnell I., Johnell O. (1996). Femoral neck geometry and radiographic signs of osteoporosis as predictors of hip fracture. *Bone*.

[16] Rubin P. J., Leyvraz P. F., Aubaniac J. M., Argenson J. N., Esteve P., De Roguin B. (1992). The morphology of the proximal femur: a three-dimensional radiographic analysis. *Journal of Bone and Joint Surgery B*.

[17] Mahaisavariya B., Sitthiseripratip K., Tongdee T., Bohez E. L., Sloten J. V., Oris P. (2002). Morphological study of the proximal femur: a new method of geometrical assessment using 3-dimensional reverse engineering. *Medical Engineering & Physics*.

[18] Synthes *Synthes Technique Guide ‘‘LCP Proximal Femoral Plate 4.5/5.0. Part of the LCP Periarticular Plating System’’*.

[19] Nishiura T., Nozawa M., Morio H. (2009). The new technique of precise insertion of lag screw in an operative treatment of trochanteric femoral fractures with a short intramedullary nail. *Injury*.

[20] Larsson S., Friberg S., Hansson L.-I. (1990). Trochanteric fractures: influence of reduction and implant position on impaction and complications. *Clinical Orthopaedics and Related Research*.

[21] Güven M., Yavuz U., Kadioğlu B. (2010). Importance of screw position in intertrochanteric femoral fractures treated by dynamic hip screw. *Orthopaedics & Traumatology: Surgery & Research*.

[22] Tannenbaum E., Kopydlowski N., Smith M., Bedi A., Sekiya J. K. (2014). Gender and racial differences in focal and global acetabular version. *Journal of Arthroplasty*.

[23] Rawal B. R., Ribeiro R., Malhotra R., Bhatnagar N. (2012). Anthropometric measurements to design best-fit femoral stem for the Indian population. *Indian Journal of Orthopaedics*.

[24] Huang J. I., Toogood P., Chen M. R., Wilber J. H., Cooperman D. R. (2007). Clavicular anatomy and the applicability of precontoured plates. *Journal of Bone and Joint Surgery A*.

[25] Tang W. M., Chiu K. Y., Kwan M. F. Y., Ng T. P., Yau W. P. (2005). Sagittal bowing of the distal femur in Chinese patients who require total knee arthroplasty. *Journal of Orthopaedic Research*.

[26] Harper M. C., Carson W. L. (1987). Curvature of the femur and the proximal entry point for an intramedullary rod. *Clinical Orthopaedics and Related Research*.

[27] Egol K. A., Chang E. Y., Cvitkovic J., Kummer F. J., Koval K. J. (2004). Mismatch of current intramedullary nails with the anterior bow of the femur. *Journal of Orthopaedic Trauma*.

[28] Chang S.-M., Song D.-L., Ma Z., Tao Y.-L., Chen W.-L., Zhang L.-Z., Wang X. (2014). Mismatch of the short straight cephalomedullary nail (PFNA-II) with the anterior bow of the femur in an asian population. *Journal of Orthopaedic Trauma*.

[29] Li K., Langdale E., Tashman S., Harner C., Zhang X. (2012). Gender and condylar differences in distal femur morphometry clarified by automated computer analyses. *Journal of Orthopaedic Research*.

[30] Bellemans J., Carpentier K., Vandenneucker H., Vanlauwe J., Victor J. (2010). The john insall award: Both morphotype and gender influence the shape of the knee in patients undergoing TKA. *Clinical Orthopaedics and Related Research*.

[31] Bolanowski W., Śmiszkiewicz-Skwarska A., Polguj M., Jédrzejewski K. S. (2005). The occurrence of the third trochanter and its correlation to certain anthropometric parameters of the human femur. *Folia Morphologica*.

[32] Bulandra A., Gielecki J. S., Leciejewska I., Karaszewski P., Sieroń D. (2003). Digital-image analysis of the femoral shaft/neck angle in human foetuses. *Folia Morphologica*.

[33] Tönnis D. (1976). Normal values of the hip joint for the evaluation of X-rays in children and adults. *Clinical Orthopaedics and Related Research*.

[34] Papaioannou T. A., Digas G., Bikos C., Karamoulas V., Magnissalis E. A. (2013). Femoral neck version affects medial femorotibial loading. *ISRN Orthopedics*.

